# Differential associations of conduct disorder, callous-unemotional traits and irritability with outcome expectations and values regarding the consequences of aggression

**DOI:** 10.1186/s13034-022-00466-x

**Published:** 2022-05-23

**Authors:** J. Elowsky, S. Bajaj, J. Bashford-Largo, R. Zhang, A. Mathur, A. Schwartz, M. Dobbertin, K. S. Blair, E. Leibenluft, D. Pardini, R. J. R. Blair

**Affiliations:** 1 Center for Neurobehavioral Research, Boys Town National Research Hospital, 14015 Flanagan Blvd. Suite #102, Boys Town, NE 68010 USA; 2 Center for Brain, Biology, and Behavior, University of Nebraska-Lincoln, Lincoln, NE USA; 3 3Department of Psychology, University of North Dakota, Grand Forks, ND USA; 4 Section on Mood Dysregulation and Neuroscience, Intramural Research Program, National Institute of Mental Health, National Institutes of Health, Bethesda, MD USA; 5 School of Criminology & Criminal Justice, Arizona State University, Phoenix, AZ USA; 6 Child and Adolescent Mental Health Centre, Mental Health Services, Capital Region of Denmark, Copenhagen, Denmark

**Keywords:** Conduct disorder, Expectations of outcomes of aggressive acts, Callous-unemotional traits, Irritability

## Abstract

**Background:**

Previous work has examined the association of aggression levels and callous-unemotional traits with outcome expectations and values regarding the consequences of aggression. Less work has examined the outcome expectations and values regarding the consequences of aggression of adolescents with Conduct Disorder (CD). Also, no studies have examined links between irritability (a second socio-affective trait associated with CD) and these social cognitive processes despite the core function of anger in retaliatory aggression and establishing dominance.

**Method:**

The current study, investigating these issues, involved 193 adolescents (typically developing [TD; *N* = 106], 87 cases with CD [*N* = 87]). Participants completed an adaptation of the Outcomes Expectations and Values Questionnaire and were assessed for CU traits and irritability via the Inventory of Callous-Unemotional traits and the Affective Reactivity Index.

**Results:**

While CD was associated with atypical outcome expectations this was not seen within statistical models including CU traits and irritability. CU traits were associated with decreased expectation that aggression would result in feelings of remorse and victim suffering, as well as decreased concern that aggressive acts would result in punishment and victim suffering. Irritability was associated with increased expectations and concern that aggression would result in dominance and forced respect.

**Conclusions:**

The results suggest that CU traits and irritability, often present in youth with CD, are associated with different forms of maladaptive outcome expectations and values regarding the consequences of aggression. This suggests that the atypical social cognitive processes underlying aggressive behavior among youth exhibiting CU traits may differ from those exhibiting problems regulating anger.

**Supplementary Information:**

The online version contains supplementary material available at 10.1186/s13034-022-00466-x.

## Introduction

Conduct disorder (CD) is a childhood disruptive behavior disorder defined by repeated and persistent antisocial behavior that involves a propensity for violating the rights of others (e.g., aggression and destruction of property) according to Diagnostic and Statistical Manual of Mental Disorders version 5 (DSM5; [1]). Patients with CD and conduct problems account for one-third to one-half of all youth referred to mental health facilities [38] and are at significant increased risk for negative social interactions, academic-related problems, and/or juvenile delinquency [18]. For this reason, extensive research has focused on the forms of maladaptive socio-affective and social cognitive processing that might underpin CD [9, 18].

Research suggests that children with conduct problems tend to exhibit an atypical social schema regarding the potential outcomes associated with engaging in aggressive behavior [29]. According to Dodge and colleagues social-information-processing model, individuals respond very rapidly to aggression cues with a sequence of mental operations that may lead to aggressive behavior in socially challenging situations [15–17]. These steps involve encoding and interpreting situational cues through attention and sensation, adopting a goal for the situation, generating possible behavioral responses and, finally, evaluating the likely positive and negative consequences of their potential responses, placing value on those consequences, and selecting an optimal response [15–17]. Here we focus on this final stage—when an individual’s behavioral choices are influenced by his/her expectations about the different outcomes (i.e., outcome expectations) and by the relative importance placed on these outcomes (i.e., outcome values) [33]. We postulate that children are more likely to behave aggressively if they believe attacking others will result in more positive than negative outcomes [15]. In line with this, studies have shown that aggressive children are less likely to expect that using aggression to resolve conflicts will result in punishment [23] and more likely to believe aggression will result in instrumental rewards and reduced aversive treatment by others ([34, 37, 39]—though see [28]). When confronting interpersonal conflict, aggressive youth place greater importance on the potential positive outcomes associated with aggression and are less concerned about the potential negative consequences. For example, youth with a history of violence are more likely to stress the importance of exacting revenge, establishing dominance, and forcing others to show them "respect" [33]. Conversely, they are less concerned that attacking provocative peers may result in punishment and victim suffering [11, 23, 33]. Taken together, studies indicate that aggressive youth exhibit both maladaptive outcome expectancies and outcome values regarding the use of aggression to deal with interpersonal conflicts.

Previous work indicates that atypical outcome expectations and values may be particularly pronounced among antisocial children and adolescents exhibiting callous-unemotional traits (CU) (i.e., lack of empathy, guilt). CU traits are elevated in patients with CD [18], associated with higher rates of delinquency [35] and proactive aggression [24] and predict a variety of antisocial outcomes [32]. Previous work has indicated that adjudicated adolescents with higher CU traits are more likely to expect that aggression will result in positive outcomes (e.g., tangible rewards, dominance) and less likely to expect it will produce negative outcomes (e.g., punishment, feelings of remorse), even after controlling for their prior history of criminal offending [36]. Similarly, studies have found that youth with higher CU traits are more concerned about establishing dominance during interpersonal conflicts [36], and less concerned that aggression could result in punishment and cause victim suffering [25, 34, 36].

As noted, CU traits are elevated in patients with CD [18] but they are not the only socio-affective trait elevated in patients with CD. Irritability is elevated in patients with CD [45]—as well as other pediatric psychiatric diagnoses including Major Depressive Disorder and Attention-Deficit/Hyperactivity Disorder [13]. Irritability is defined as an “increased propensity to exhibit anger relative to one’s peers” [26], p. 277) and a “relative dispositional tendency to respond with anger to blocked goal attainment, and includes both mood (trait) and behavioral (reactive state) dysregulation” [14, 19], p. 69,see also; [41, 44]. Previous work examining neuro-cognitive correlates of CU traits and irritability in patients with CD has indicated that reduced differential signalling of reward relative to punishment feedback is particularly associated with CU traits rather than irritability [45]. In contrast, heightened threat responsiveness is particularly associated with irritability and may be moderated by level of CU traits [45]. Therefore, rritability may be important to consider with respect to outcome expectations/values related to aggression. Irritability is highly correlated with reactive aggression and theoretical positions on irritability/reactive aggression stress the interrelationship between these constructs [8, 27]. Irritability can be triggered by frustration, perceived threat and social provocation [5, 8, 27]. The relationship with social provocation is particularly interesting in the current context. A major reason for anger following social provocation is to re-establish dominance i.e., the response to an unfair allocation is based on the individual’s desire to establish at least equality with the allocator [2, 6, 8]. However, the relationship between irritability with social goals/expectations and aggression has received very little attention.

In short, previous work in forensic and community samples shows that aggressive youth exhibit atypical outcome expectancies and outcome valuations with respect to aggressive acts and that this atypical social information processing may be particularly marked in youth with CU traits (e.g., [25, 34, 36]. However, there has been less work with clinical cases and patients with CD. Furthermore, we are unaware of work examining the association of irritability with atypical outcome expectancies and outcome valuations regarding aggressive acts. Elevated CU traits and irritability co-occur, particularly in cases with CD (e.g., [45], yet the neuro-cognitive abnormalities associated with CU traits and irritability differ. CU traits have been associated with reduced responses to distress cues [4, 8, 31, 43]. In contrast, irritability is associated with increased responsiveness to threat and frustration and dysfunction in systems engaged in response control [8, 19, 27]. Given the differential neuro-cognitive abnormalities associated with CU traits and irritability, it is important to test whether they are associated with differential forms of atypical outcome expectancies and outcome valuations regarding aggressive actions (see also above). We investigate this issue in 194 adolescents who completed a version of the Outcome Expectations and Values Questionnaire [36]. Based on previous studies, we predicted that CD and higher levels of CU traits would be associated with lack of guilt or concern for victim suffering, and greater concern for status. Based on theories on irritability and anger [2, 6, 8], we predicted that higher levels of irritability would be positively associated with greater expectations of establishing dominance following aggression and concern for status.

## Methods and materials

### Participants

Participants included 193 youths aged 10–18 from both a residential treatment program and the surrounding community (87 from the residential treatment program and 106 from the community); average age = 14.63 (*SD* = 2.41), average IQ = 104.75 (*SD* = 12.76), 112 males. Participants were recruited from a broader study on neuro-cognitive correlates in youth with behavioral and emotional problems; i.e., CD, Attention Deficit Hyperactivity Disorder [ADHD], Major Depressive Disorder [MDD]) and Generalized Anxiety Disorder [GAD]. Participants were included in this study if they met diagnostic criteria for CD (*N* = 87) or if they met criteria for no psychiatric condition (i.e., typically developing [TD]; *N* = 106).

Youths recruited from the residential treatment program had been referred for behavioral and mental health problems and were recruited shortly after their arrival. Participants from the community were recruited through flyers and social media and included youth with psychopathology and TD youth. Clinical characterization of all participants was done through psychiatric interviews by licensed and board-certified child and adolescent psychiatrists with the participants and their parents, to adhere closely to common clinical practice (see Additional file 1: for exclusion criteria).

The Boys Town National Research Hospital institutional review board approved this study. A doctoral level researcher or a member of the clinical research team obtained written informed consent and assent. In all cases, youth had the right to decline participation at any time before or during the study.

### Measures

#### Task: *the Outcome Expectations and Values Questionnaire (OVQ; *[36]

Participants completed a slightly shortened version of the Outcome Expectations and Values Questionnaire [36]. The measure consisted of seven short vignettes in which the participant is asked to imagine scenarios in which they are provoked by a same-sex peer (e.g., ‘Another teen trips you while you’re walking down the hall') and respond to this provocation via a specified aggressive act. This specified aggressive act differs by vignette and involves engaging in verbal (e.g., threating to hit the person), physical (e.g., hitting them), or relational (e.g., posting something embarrassing about them online) aggression.

To assess outcome expectations, the participant was asked to rate the likelihood (i.e.,) on a four-point scale (1 = "Definitely NO!" to 4 = "Definitely YES!") that four different outcomes would occur following their aggressive act. The outcomes were: (i) feelings of remorse (‘You would feel bad or guilty about what you did’); (ii) victim suffering (‘He/She would feel hurt or scared’); (iii) dominance (‘It would let him/her know who’s in charge or boss’); and (iv) forced respect (‘It would make him/her show you some respect’). Responses to each of these outcomes were averaged across the seven vignettes. Participants showed strong internal consistency regarding their rating of the likelihood of the four different outcomes across the 7 vignettes (for feelings of remorse [α = 0.92], victim suffering [α = 0.81], dominance [α = 0.86], and forced respect [α = 0.87]).

To assess outcome values, participants were asked to rate how much they would care on a four-point scale (1 = ‘Would not care at all’ to 4 = ‘Would care a lot’) about four different outcomes occurring as the result of their engaging in the aggressive act. The outcomes were punishment (‘You got caught and were punished’), victim suffering (‘He/She felt bad and wanted to cry’), dominance (‘He/She recognized that you were in charge or boss’), and forced respect (‘He/She showed you some respect’). Responses to each of these outcomes were averaged across the seven vignettes. Participants showed strong internal consistency regarding how much they would care about the four different outcomes across the 7 vignettes (for punishment [α = 0.92], victim suffering [α = 0.93], dominance [α = 0.92] and forced respect [α = 0.93]).

##### Symptom

CU traits were assessed via the *Inventory of Callous-Unemotional Traits* (ICU: [20], a 24-item self-report questionnaire with excellent psychometric properties, including internal consistency (α = 0.77), and test–retest reliability [20]. Irritability was assessed via the *Affective Reactive Index* (ARI: [41], a seven-item self-report questionnaire with excellent internal consistency (α = 0.90) and test–retest reliability [41]. Note that ICU items are focused on identifying level of guilt/remorse/empathy (e.g., “I feel bad or guilty when I do something wrong” [inverse scored]), *reduced* affect (e.g., “I do not show my emotions to others”), and/or lack of concern about performance (e.g., “work hard on everything I do” [inverse scored]). ARI items, in contrast, are focused on identifying level of anger propensity (e.g., “I often lose my temper”). There is no item level overlap between the ICU and ARI.

Conduct problems were assed via the parent-report conduct problems subscale of the *Strengths and Difficulties Questionnaire* (SDQ; [22]).

### Statistical analyses

ARI scores showed high levels of skewness and kurtosis. To reduce the possibility of outlier scores having a disproportionate impact on the data, Rankit Transformations were applied to participants' ICU and ARI scores (*pre-transformation—*skewness: ICU = 0.54, ARI = 1.81; kurtosis: ICU = 0.14, ARI = 2.88; *post-transformation—*skewness: ICU = 0.02, ARI = 0.54; kurtosis: ICU = − 0.16, ARI = − 0.57). The Rankit-Transformed ARI and ICU scores were used as continuous covariates in analyses.

#### Clinical data

Using independent sample *t*-tests, we assessed group differences (participants with CD relative to TD participants) in age, IQ, sex, ICU, ARI and SDQ-CP. We used correlational analyses to examine association of ICU and ARI (raw scores) with demographic and clinical variables. For these analyses, presence of diagnosis (CD, ADHD, MDD and GAD) and prescribed use of a drug class (antipsychotic, stimulant and SSRI medications) was coded as 1, absence of diagnosis/prescribed use coded as 0. Steiger z-tests [40] were performed to compare the relative strength of the correlations between ICU versus ARI scores and these variables.

#### Task data

##### Correlational analyses

Initial analyses focused on potential replication of previous work. Specifically, initial correlation analyses were conducted examining the associations between CD diagnostic status, ICU and ARI scores, age, sex and IQ and the outcome expectation and values ratings.

##### Group differences

Group differences for responses to the 8 task questions (4 ratings of “likelihood”, 4 ratings of “caring”) were examined using a 2 (Group: Conduct Disorder, Typically Developing) × 2 (Sex: Male, Female) × 8 (Task Question) MANCOVA with age and IQ as covariates. This analysis was designed to determine the extent to which CD diagnostic status was associated with atypical outcome expectations and outcome values.

##### ICU/ARI scores

The MANCOVA described above was repeated with two additional covariates: transformed ICU/ARI scores. This analysis was designed to test for differential associations of ICU and ARI scores with specific outcome expectations and values. Second, given that CU traits and irritability are important components of CD, this analysis tests the extent to which addition of ICU/ARI score covariates removed the association of CD with atypical outcome expectations and outcome values.

#### Follow-up analyses

##### Raw score analysis

Since transformation of the ICU/ARI scores might alter relationships between underlying neuro-cognitive dysfunction, the ICU/ARI MANCOVA was repeated using raw rather than transformed ICU/ARI scores.

#### Potential prescribed medication confounds

If medication prescriptions were significantly more related to ICU than ARI scores (or vice versa), our main MANCOVA was repeated following the removal of all participants prescribed these medications.

For all analyses, effect sizes (partial etas) for observed findings are reported.

## Results

### Clinical data

#### Group differences

As expected, participants with CD scored significantly higher than TD participants on the ARI, ICU and SDQ-CP and also age and IQ though not sex; see Table 1. Within the sample, CU traits and irritability showed significant correlations with age, IQ, sex and each other. However, Steiger z-tests indicated that the association strength of these variables with ICU versus ARI scores was not significantly different (except for prescription of antipsychotic medications and sex); see Table 1.

**Table 1:** Participant characteristics by group

	CD		TD		*F* (1,192)	*p* value	Correlation with ARI	Correlation with ICU	Steiger's Z	*p* value
	Mean	SD	Mean	SD						
Age	15.77	1.77	13.71	2.47	41.57	< 0.001	0.18*	0.31**	− 1.74	= 0.08
IQ	100.30	11.92	108.4	12.31	21.32	< 0.001	− 0.11	− 0.17*	0.79	= 0.43
ARI	3.64	3.53	0.98	1.34	50.06	< 0.001	–	0.43**		
ICU	25.46	8.03	15.51	6.61	77.81	< 0.001	0.43**	–		
SDQ-CP	6.74	1.96	0.33	0.66	897.70	< 0.001	0.47**	0.51**	− 0.65	= 0.51
RPQ Total score	12.04	7.73	4.43	3.11	80.97	< 0.001	0.74**	0.58**	3.17	= 0.002
RPQ-Reactive	9.12	5.52	4.10	2.82	63.82	< 0.001	0.73**	0.52**	4.03	< 0.001
RPQ-Proactive	2.96	2.97	0.37	0.80	70.97	< 0.001	0.58**	0.56**	0.27	= 0.79
Conners (ADHD)	8.75	6.11	0.45	1.78	177.33	< 0.001	0.37**	0.36**	0.14	= 0.89
Sex	0.64	0.48	0.53	0.50	2.62	= 0.11	− 0.11	0.19*	− 3.84	< 0.001
CD	87	–	0	–	–	–	0.46**	0.56**	− 1.64	= 0.10
ODD	75	–	0	–	–	–	0.52**	0.54**	− 0.33	= 0.37
ADHD	64	–	0	–	–	–	0.45**	0.42**	0.46	= 0.64
MDD	20	–	0	–	–	–	0.43**	0.41**	0.37	= 0.71
GAD	30	–	0	–	–	–	0.43**	0.35**	1.20	= 0.23
Antipsychotic	6	–	0	–	–	–	0.09	0.28**	− 2.56	= 0.01
Stimulant	18	–	0	–	–	–	0.21**	0.13	1.02	= 0.31
SSRI	15	–	0	–	–	–	0.22**	0.21**	0.17	= 0.86

*CD* Conduct Disorder, *TD* Typically developing, *SD* Standard deviation, *ARI* Affective Reactivity Index (raw score), *ICU* Inventory of Callous Unemotional Traits (raw score), *SDQ-CP* Strengths and Difficulties Questionnaire—conduct problems subscale, *RPQ* Reactive-Proactive Questionnaire, *ADHD* Attention Deficit Hyperactivity Disorder, *MDD* Major Depressive Disorder, *GAD* Generalized Anxiety Disorder

^*^significant at p < 0.05, **significant at p < 0.001

### Task data

#### Correlational analyses

Our initial correlation analyses revealed significant associations between CD diagnostic status (with or without a CD diagnosis), SDQ-CP, ICU and ARI scores and demographic variables and both outcome expectations and outcome values (specifically, valuations of potential punishment and victim suffering); see Table 2.

**Table 2:** Associations of CD diagnostic status, irritability, ICU traits and demographic variables with question responses (questions are paraphrased)

	CD	SDQ-CP	ARI	ICU	Age	Male	IQ
Outcome expectations							
Guilt	− 0.52**	− 0.481**	− 0.27**	− 0.47**	− 0.42**	− 0.38**	0.23**
Victim suffering	− 0.20**	− 0.160*	0.004	− 0.26**	− 0.14	− 0.29**	0.03
Dominance	0.20**	0.189*	0.24**	0.22**	0.19**	0.20**	− 0.12
Forced respect	0.13	0.121	0.16*	0.18*	0.12	0.15*	− 0.14
Outcome values							
Punishment	− 0.48**	− 0.424**	− 0.24**	− 0.39**	− 0.38**	− 0.28**	0.17*
Victim suffering	− 0.51**	− 0.458**	− 0.19**	− 0.49**	− 0.45**	− 0.38**	0.22**
Dominance	0.05	0.043	0.18*	0.10	− 0.04	0.14	− 0.04
Force respect	− 0.02	− 0.023	0.15*	− 0.06	− 0.01	0.06	− 0.04

*CD* Conduct Disorder, *SDQ-CP* Strengths and Difficulties Questionnaire—conduct problems subscale, *ARI* Affective Reactivity Index (raw score), *ICU* Inventory of Callous Unemotional Traits (raw score)

^*^significant at p < 0.05, **significant at p < 0.001

#### Group differences

The MANCOVA on group differences in task response was highly significant for group [*F* = 5.183, *p* < 0.001; pη^2^ = 0.190] and also sex and age [*F* = 4.199 & 2.299, *p* < 0.001 & = 0.023; pη^2^ = 0.160 & 0.094 respectively]. It was not significant for IQ [*F* = 0.579, *p* = 0.794; pη^2^ = 0.025]. With respect to individual task items, compared to individuals without CD, those with CD showed lower expectations of experiencing guilt and that the victim would experience distress. They also cared less about possible punishment (Q5) or the victim’s distress (Q6); see Table 3.

**Table 3:** Group-based MANCOVA results, group means, and standard deviations by question (questions are paraphrased)

	Outcome expectations				Outcome values			
	Guilt	Victim suffering	Dominance	Forced respect	Punishment	Victim suffering	Dominance	Forced respect
Group-based MANCOVA results by question								
Group								
F	**35.01**	**4.77**	2.02	0.27	**26.04**	**30.65**	0.76	0.04
*p*	0.00	0.03	0.16	0.60	0.00	0.00	0.38	0.85
pη^2^	0.16	0.03	0.01	0.00	0.12	0.14	0.00	0.00
Sex								
F	**25.44**	**12.70**	**6.36**	**4.72**	**11.81**	**23.92**	**4.50**	0.91
*p*	0.00	0.00	0.01	0.03	0.00	0.00	0.04	0.34
pη^2^	0.12	0.07	0.03	0.03	0.06	0.12	0.02	0.01
Age								
F	**6.67**	0.05	0.85	0.12	**5.75**	**10.32**	2.33	0.29
*p*	0.01	0.82	0.36	0.73	0.02	0.00	0.13	0.59
pη^2^	0.04	0.00	0.01	0.00	0.03	0.05	0.01	0.00
IQ								
F	0.50	0.34	0.94	2.53	0.00	0.50	0.87	0.71
*p*	0.48	0.56	0.33	0.11	1.00	0.48	0.35	0.40
pη^2^	0.00	0.00	0.01	0.01	0.00	0.00	0.01	0.00
Group means and standard deviations by question								
CD								
Mean	2.37	2.70	2.32	2.42	2.59	2.28	2.15	2.51
SD	0.78	0.55	0.55	0.60	0.88	0.87	0.82	0.84
TD								
Mean	3.22	2.92	2.09	2.26	3.41	3.22	2.07	2.55
SD	0.62	0.52	0.57	0.58	0.63	0.73	0.80	0.86

*CD* Conduct disorder, *TD* Typically developing

Bolded numbers in the MANCOVA table indicate significant results (*p* < 0.05)

### ICU/ARI scores

In a MANCOVA including the ICU and ARI as covariates, group was no longer significant [*F* = 1.708, *p* < 0.101; pη^2^ = 0.085]. However, both ICU and ARI were significant [*F* = 3.078 and 2.502, *p* = 0.003 and = 0.014; pη^2^ = 0.143 and 0.119 respectively] (N.B.: homogeneity of slopes was tested and there were no significant interactions between CD status and either ICU or ARI scores; F = 0.504 and 0.777, P = 0.852 and 0.624 respectively). The influences of sex and age remained [*F* = 3.361 and 2.239, *p* = 0.001 and = 0.028; pη^2^ = 0.154 and 0.108 respectively] while those of IQ remained non-significant [*F* = 0.259, *p* = 0.978; pη^2^ = 0.014]. ICU scores were negatively associated with expecting to feel guilty, expecting the victim to experience distress, and concern about possible punishment and about the victim’s distress. They were not associated with responses to the other items (see Table 4). ARI scores were positively associated with both expectations that the aggressive act would establish dominance and force respect (Q4) and with placing a high value on the established dominance and forced respect. They were not associated with responses to the other items (see Table 4).

**Table 4:** Results for the MANCOVA including the ICU and ARI covariates by question (questions are paraphrased)

	Outcome expectations				Outcome values			
	Guilt	Victim suffering	Dominance	Forced respect	Punishment	Victim Suffering	Dominance	Forced respect
Group-based MANCOVA results by question								
CD								
*F*	**7.63**	2.45	0.54	1.58	**5.63**	**8.22**	0.42	0.36
*p*	0.01	0.12	0.46	0.21	0.02	0.01	0.52	0.55
pη^2^	0.05	0.02	0.00	0.01	0.04	0.05	0.00	0.00
sICU								
*F*	**5.61**	**5.16**	0.28	0.62	**4.00**	**9.46**	0.00	3.86
*p*	0.02	0.03	0.60	0.43	0.05	0.00	0.96	0.05
pη^2^	0.04	0.03	0.00	0.00	0.03	0.06	0.00	0.02
ARI								
*F*	0.90	3.84	**8.49**	**4.89**	0.47	0.55	**5.22**	**8.89**
*p*	0.34	0.05	0.00	0.03	0.49	0.46	0.02	0.00
pη^2^	0.01	0.02	0.05	0.03	0.00	0.00	0.03	0.05
Sex								
*F*	**20.86**	**5.29**	**7.59**	3.78	**7.23**	**14.40**	**6.20**	3.83
*p*	0.00	0.02	0.01	0.05	0.01	0.00	0.01	0.05
pη^2^	0.12	0.03	0.05	0.02	0.05	0.09	0.04	0.02
Age								
*F*	**6.64**	0.03	3.26	1.88	**5.11**	**10.16**	0.66	0.00
*p*	0.01	0.86	0.07	0.17	0.03	0.00	0.42	0.99
pη^2^	0.04	0.00	0.02	0.01	0.03	0.06	0.00	0.00
IQ								
*F*	0.11	0.18	0.25	0.57	0.00	0.00	0.12	1.10
*p*	0.74	0.68	0.62	0.45	1.00	0.96	0.73	0.30
pη^2^	0.00	0.00	0.00	0.00	0.00	0.00	0.00	0.01

*CD* Conduct Disorder, *ICU* Inventory of Callous Unemotional Traits, *ARI* Affective Reactivity Index

Bolded numbers in the MANCOVA table indicate significant results (*p* < 0.05)

#### Follow-up analyses

##### Raw score analysis

A third MANCOVA including raw, rather than transformed, ICU and ARI scores revealed very similar results to the MANCOVA described above (see Additional file 2: Table S1 and S2).

##### Potential prescribed medication confounds

Prescription of antipsychotic medications showed a stronger association with ICU than ARI scores (see Table 1). Therefore, our main MANCOVA was repeated following the removal of participants who had been prescribed medications. This MANCOVA revealed very similar results to the MANCOVA described above (see Additional file 2: Table S1 and S2).

## Discussion

The current study examined associations of CD diagnostic status, CU traits and irritability with participants’ social perceptions of peer conflicts. Initial correlation analyses revealed that both CD diagnostic status and CU traits were negatively associated with expectations of feeling guilty and causing victim distress, and with placing importance on possible punishment or the victim’s distress. These associations were also seen with irritability (ARI scores). However, statistical models accounting for the influence of ICU, ARI, and CD diagnosis indicated selective associations among ICU, ARI, and specific forms of social perception. Specifically, CD diagnostic status and CU traits were negatively associated with expectations of feeling guilty and causing victim distress, and with placing value on possible punishment or the victim’s distress. In contrast, irritability (as indexed by the ARI) was positively associated with expectations that the aggressive act would establish dominance and force respect and with placing value on establishing dominance and forcing respect. Notably, associations of CD diagnosis with task performance were accounted for by group differences in CU traits and irritability.

CD diagnostic status and levels of CU traits and irritability are significantly correlated (in the current study, r = 0.43 or greater; see Table 1). CU traits have long been linked with CD [10, 18, 21, 24, 30, 35, 42]. More recently, the importance of irritability in CD has been stressed (e.g., [8]. While they are inter-correlated, CU traits and irritability are associated with different forms of atypical neuro-cognitive functioning (relating to responsiveness to distress cues, threat responsiveness, response control and reinforcement-based decision-making; e.g., [8, 13, 45]. These forms of atypical neuro-cognitive functioning have all been all associated with CD [8, 18]. Indeed, it can be argued that the pathophysiology underpinning CU traits and irritability largely underpins the presentation of CD [8]. Consistent with this, while the regression and group focused MANCOVA revealed associations of CD diagnostic status with task performance, these were removed following the addition of the CU trait and irritability scores.

As stated above, previous findings indicate that higher CU traits and levels of aggression are negatively associated with expectations of guilt and victim distress and concern with potential punishment and victim distress [11, 34] and positively associated with expectations that aggressive responses would establish dominance and engender forced respect [29, 33]. These findings were replicated in our initial correlation analyses. However, they were also seen with respect to irritability. Yet, as noted above, data indicate that CU traits and irritability are associated with different forms of atypical neuro-cognitive functioning [8]. Given the significant positive correlation between CU traits and irritability, this raised the possibility that co-occurring dysfunction might give rise to spurious associations. Note that while the significant positive correlation between CU traits and irritability might give rise to spurious associations between CU/ARI scores and motivations/outcome expectances, this does not mean that the association between CU and ARI scores is spurious. We believe, and the current data support, the contention that the neuro-cognitive abnormalities underpinning CU and ARI scores are dissociable (see also [8]. But CU traits and irritability do often co-occur – at least in the case of patients with CD [8]. The exact reasons for this remain unknown but might reflect common genetic or social etiological factors.”

Our MANCOVA analyses revealed that CU traits, in particular, were negatively associated with expectations of experiencing guilt and victim’s distress and with caring about either potential punishment or the victim’s distress. This finding was consistent with predictions and previous work [11, 33, 34]. As such, these data are compatible with views that CU traits reflect a specific form of neuro-cognitive dysfunction relating to reduced emotional responsiveness to the distress of others, expressed as reduced guilt, empathy and concern for victims [3, 7, 42]. Individuals who are are less emotionally responsive to the distress of other individuals may be likely to initiate actions that will harm others in order to achieve their goals [3, 7, 42]. Given the overlap in neural circuitry between systems responsive to distress cues and systems responsive to threatening stimuli (e.g., the amygdala) [8], CU traits are also thought to relate to reduced threat responsiveness that can manifest as a reduced concern about potential punishment.

The lack of an association between CU traits and a perception that aggressive responses would engender dominance and force respect and concerns about status and perceived respect could be considered to be unexpected. Previous work has reported that CU traits were positively related to these perceptions [33, 34, 36]. However, neither empathy based models of CU traits (cf. Ref. [3, 7, 42] nor other views (cf. Ref. [21] have provided adequate accounts of this association. If considered at all, these associations are considered secondary consequences of the empathy deficit (cf. Ref. [3, 7]; i.e., the individual shows greater concern with establishing dominance because they are indifferent to the distress of the dominated individual. Here, we use a regression analysis to replicate the association between CU traits and a perception that aggressive responses engender dominance and force respect. However, inclusion of irritability scores in the MANCOVA analysis, indicated that it was irritability, rather than CU traits, that was particularly associated with perceptions that aggressive responses would engender dominance and force respect. Possibly, previous reports relating CU to dominance and forced respect, may primarily reflect the pathology of frequently co-occurring irritability. Additional work will be needed to investigate this possibility.

As noted, the MANCOVA analysis indicated that irritability was positively associated with expectations that aggression would engender dominance and force respect and valuing dominance and forced respect. This is interesting as theoretical accounts of the communicatory value of anger suggest that a major goal of the display is to re-establish dominance—the response to an unfair allocation is based on the individual’s desire to establish at least equality with the allocator [2, 6, 8]. Consistent with this, the current data indicate that individuals who are more prone to anger (i.e., have higher irritability/ARI scores) are predisposed to focus on the potential for establishing dominance over, and respect from, those aggressed against during social conflict.

Placing these results within the social information processing framework [15–17], our data suggest that the differential forms of atypical neuro-cognitive functioning associated with CU traits and irritability may have different influences on the final stage of processing i.e., evaluating the likely positive and negative consequences of their potential responses. ICU scores were associated with reduced concern about both possible punishment and the victim’s distress. This potentially reflects the reduced threat processing (e.g., [12] and responsiveness to the distress of others [8] seen in individuals with elevated CU traits; the reduced emotional responses would reduce the (negative) value associated with possible punishment and the victim’s distress. Unsurprisingly, given that a major component of CU traits is reduced guilt/empathy [21], ICU scores were negatively associated with the expectation of feeling guilt. More interestingly, CU traits were also associated with a reduced expectancy that the victim would feel distress. Potentially, the reduced emotional response to the distress of others reduces the salience of this distress and leads to the individual being less likely to consider that the victim will be distressed. Irritability was, in contrast, associated with an increase in value of outcomes involving the establishment of dominance and the enforcement of respect. Irritability has been related to heightened sensitization of systems mediating anger [8, 13, 27]. A major reason for displaying anger following social provocation is to re-establish dominance [2, 6, 8]. The data here suggest that irritability is not only associated with an increase in the likelihood of displaying anger but also a (positive) change in the value of outcomes (establishment of dominance and the enforcement of respect) associated with anger. It also appears that irritability, perhaps reflecting experience, increases the judged probability that a potential aggressive response would reestablish dominance and force respect.

Five caveats should be noted with respect to the current study. First, prescription of antipsychotic medications was significantly more related to ICU than ARI scores. As such, the current results might reflect medication usage. However, subsequent analyses following the removal of medicated participants yielded similar results to the main analysis. Second, ICU and ARI scores were transformed before inclusion in our principle MANCOVA. Given the significant skewness/kurtosis of the ARI scores, this was done to reduce the possibility of outlier participants over-contributing to the results. Importantly, repetition of the analysis using raw ICU and ARI scores rather than transformed scores yielded similar results. Third, many of the adolescents with CD also met diagnostic criteria for ADHD, MDD and GAD. As such, pathology associated with ADHD, MDD and/or GAD symptoms might underpin some of the group differences in Outcomes measures reported in Table 3. This is a consistent concern for group-based analyses of conditions with high levels of co-morbid conditions (it is possible to select participants only with the target condition however such patients are unlikely to reflect the clinical norm of patients with the target condition). Importantly, though dimensional analyses can be less prone to these concerns. As can be seen in Table 1, there were no significant differences in the strengths of the correlations of ICU vs ARI scores with ADHD, MDD and/or GAD diagnostic status. As such, differential effects of ICU and ARI in their associations with Outcome dependent measures are unlikely to reflect pathology underpinning ADHD, MDD and/or symptoms. Fourth, participants’ motivations and outcome expectancies were measured by self-report. As such, the task assesses the participant’s self-concepts of their motivations/outcome expectancies. Fifth, diagnoses followed clinical practice rather than the implementation of a structured or semi-structured diagnostic interview. While this could raise concerns regarding the CD diagnoses, it is important to note that: (i) these diagnoses were supported by the SDQ conduct problems scores; and (ii) the CD symptoms of the adolescents with CD diagnoses were sufficiently severe to warrant residential care.

In conclusion, CD diagnostic status and CU traits show significant negative associations with likelihood of experiencing guilt or empathy and concern about either potential punishment or the victim’s distress during social conflict circumstances. Indeed, the relationships with CU traits, and the relationship between CD diagnostic status and CU traits, effectively explained these relationships between CD diagnostic status and task performance. Irritability, in contrast, was particularly related to a focus on the potential for establishing dominance over, and respect from, those aggressed against during social conflict.

## Supplementary Information

**Additional file 1.** Exclusion criteria, Rationale for analyzing CD as a categorical measure and ICU and ARI as continuous measures and Rationale for including age and IQ as covariates and sex as a group variable in the MANCOVAs.

**Additional file 2: Table S1.** Results of MANCOVAs involving ICU and ARI raw scores and excluding participants prescribed antipsychotic medications. **Table S2.** Results for the MANCOVAs involving ICU and ARI raw scores and excluding participants prescribed antipsychotic medications by question (questions are paraphrased). Bolded numbers in the MANCOVA table indicate significance. **Table S3.** Results of group-based MANCOVAs including MDD and GAD diagnostic status as fixed factors. **Table S4.** Results for the group-based MANCOVA including MDD and GAD diagnostic status as fixed factors by question (questions are paraphrased). Bolded numbers in the MANCOVA table indicate significance.

## Data Availability

The datasets during and/or analysed during the current study available from the corresponding author on reasonable request.
