# Supplementary material for: Differential associations of conduct disorder, callous-unemotional traits and irritability with outcome expectations and values regarding the consequences of aggression
Source: Child Adolesc Psychiatry Ment Health. 2022 May 23;16:38. doi: 10.1186/s13034-022-00466-x (PMC9128221; doi:10.1186/s13034-022-00466-x)
Supplement: Supplementary file 1 — Additional file 1. Exclusion criteria, Rationale for analyzing CD as a categorical measure and ICU and ARI as continuous measures and Rationale for including age and IQ as covariates and sex as a group variable in the MANCOVAs. [file 13034_2022_466_MOESM1_ESM.docx]

**SUPPLEMENTAL INFORMATION**

Exclusion criteria:

Exclusion criteria for the broader project included IQ<75 assessed with the Wechsler Abbreviated Scale of Intelligence (WASI two-subtest form; Wechsler, 2011), non-psychiatric medical conditions requiring the use of medication with psychotropic effects (e.g., beta blockers, steroids), current psychosis, pervasive developmental disorders, Tourette’s disorder, neurological disorders. Current psychiatric conditions (other than psychotic disorders or pervasive developmental disorders) were not exclusionary for the participants with CD. Use of psychotropic medications for psychiatric indications (e.g., stimulants, selective serotonin reuptake inhibitors, and anti-psychotics) was not exclusory.

Rationale for analyzing CD as a categorical measure and ICU and ARI as continuous measures:

CD is analyzed as a categorical measure because the diagnosis is categorical.

Our theoretical view is that there are neuro-cognitive mechanisms that, when dysfunctional, give rise to the behavioral dimensions of CU traits and irritability (e.g., Blair, 2018). As such we index CU traits and irritability as continuous measures (ICU and ARI scores).

Rationale for including age and IQ as covariates and sex as a group variable in the MANCOVAs:

Age and IQ were included as covariates in the MANCOVAs as the participants with CD were significantly older and had lower IQ than the typically developing participants (see Table 1).

Sex was included as a group variable as the correlational analyses showed that sex was significant associated with outcome expectations and values (see Table 2).

Supplemental Table 1: Results of MANCOVAs involving ICU and ARI raw scores and excluding participants prescribed antipsychotic medications.

Supplemental Table 2: Results for the MANCOVAs involving ICU and ARI raw scores and excluding participants prescribed antipsychotic medications by question (questions are paraphrased). Bolded numbers in the MANCOVA table indicate significance.

Supplemental Table 3: Results of group-based MANCOVAs including MDD and GAD diagnostic status as fixed factors.

Supplemental Table 4: Results for the group-based MANCOVA including MDD and GAD diagnostic status as fixed factors by question (questions are paraphrased). Bolded numbers in the MANCOVA table indicate significance.
