# Supplementary material for: Differential associations of conduct disorder, callous-unemotional traits and irritability with outcome expectations and values regarding the consequences of aggression
Source: Child Adolesc Psychiatry Ment Health. 2022 May 23;16:38. doi: 10.1186/s13034-022-00466-x (PMC9128221; doi:10.1186/s13034-022-00466-x)
Supplement: Supplementary file 2 — Additional file 2: Table S1. Results of MANCOVAs involving ICU and ARI raw scores and excluding participants prescribed antipsychotic medications. Table S2. Results for the MANCOVAs involving ICU and ARI raw scores and excluding participants prescribed antipsychotic medications by question (questions are paraphrased). Bolded numbers in the MANCOVA table indicate significance. Table S3. Results of group-based MANCOVAs including MDD and GAD diagnostic status as fixed factors. Table S4. Results for the group-based MANCOVA including MDD and GAD diagnostic status as fixed factors by question (questions are paraphrased). Bolded numbers in the MANCOVA table indicate significance. [file 13034_2022_466_MOESM2_ESM.docx]

Table S1: Results of MANCOVAs involving ICU and ARI raw scores and excluding participants prescribed antipsychotic medications.

| **MANCOVA: Involving ICU and ARI raw scores** | ***F* (8,148)** | ***p* value** |
| --- | --- | --- |
| **Group** | 1.80 | 0.08 |
| **Sex** | 3.47 | 0.001 |
| **Age** | 2.18 | 0.03 |
| **IQ** | 0.20 | 0.99 |
| **ICU** | 3.05 | 0.003 |
| **ARI** | 2.45 | 0.02 |
| **MANCOVA: Excluding participants prescribed antipsychotic medications** | ***F* (8,143)** | ***p* value** |
| **Group** | 1.78 | 0.09 |
| **Sex** | 3.02 | 0.004 |
| **Age** | 2.08 | 0.04 |
| **IQ** | 0.37 | 0.94 |
| **ICU** | 2.89 | 0.005 |
| **ARI** | 2.40 | 0.02 |

Note: ICU: Inventory of Callous Unemotional Traits; ARI: Affective Reactivity Index.

Table S2: Results for the MANCOVAs involving ICU and ARI raw scores and excluding participants prescribed antipsychotic medications by question (questions are paraphrased). Bolded numbers in the MANCOVA table indicate significance.

|  |  | **Outcome Expectations** | | | | | **Outcome Values** | | | | | | | |  |
| --- | --- | --- | --- | --- | --- | --- | --- | --- | --- | --- | --- | --- | --- | --- | --- |
|  |  | **Guilt** | **Victim Suffering** | **Dominance** | | **Forced Respect** | | **Punishment** | | **Victim Suffering** | | **Dominance** | | **Forced Respect** | |
| **Results for ICU and ARI of MANCOVA involving ICU and ARI raw score covariates** | | | | | | | | | | | | | | |  |
| **ICU** | *F* | **4.855** | **4.967** | 0.449 | 0.575 | | **4.829** | | **10.968** | | 0.001 | | 3.962 | |  |
|  | *p* | 0.029 | 0.027 | 0.504 | 0.449 | | 0.029 | | 0.001 | | 0.972 | | 0.048 | |  |
|  | pη^2^ | 0.03 | 0.031 | 0.003 | 0.004 | | 0.03 | | 0.066 | | 0 | | 0.025 | |  |
| **ARI** | *F* | 1.266 | 3.63 | **7.59** | **6.22** | | 0.071 | | 1.113 | | **4.478** | | **8.546** | |  |
|  | *p* | 0.262 | 0.059 | 0.007 | 0.014 | | 0.79 | | 0.293 | | 0.036 | | 0.004 | |  |
|  | pη^2^ | 0.008 | 0.023 | 0.047 | 0.039 | | 0 | | 0.007 | | 0.028 | | 0.052 | |  |
| **Results for ICU and ARI of MANCOVA excluding participants prescribed antipsychotic medications** | | | | | | | | | | | | | | |  |
| **ICU** | *F* | **4.376** | **4.39** | 0.378 | 0.829 | | **2.061** | | **6.954** | | 0.123 | | 2.847 | |  |
|  | *p* | 0.038 | 0.038 | 0.54 | 0.364 | | 0.153 | | 0.009 | | 0.726 | | 0.094 | |  |
|  | pη^2^ | 0.028 | 0.028 | 0.003 | 0.005 | | 0.014 | | 0.044 | | 0.001 | | 0.019 | |  |
| **ARI** | *F* | 0.977 | 3.5 | **7.683** | **4.113** | | 0.642 | | 0.3 | | **4.721** | | **7.99** | |  |
|  | *p* | 0.325 | 0.063 | 0.006 | 0.044 | | 0.424 | | 0.585 | | 0.031 | | 0.005 | |  |
|  | pη^2^ | 0.006 | 0.023 | 0.049 | 0.027 | | 0.004 | | 0.002 | | 0.031 | | 0.051 | |  |

Note: ICU: Inventory of Callous Unemotional Traits; ARI: Affective Reactivity Index.

Table S3: Results of group-based MANCOVAs including MDD and GAD diagnostic status as fixed factors.

| **MANCOVA:** | ***F* (8,148)** | ***p* value** |
| --- | --- | --- |
| **Group** | 4.35 | 0.000 |
| **Sex** | 0.45 | 0.89 |
| **Age** | 2.03 | 0.09 |
| **IQ** | 0.55 | 0.82 |
| **GAD** | 0.96 | 0.47 |
| **MDD** | 0.53 | 0.83 |

Note: GAD: Generalized Anxiety Disorder; MDD: Major Depressive Dsiorder

Table S4: Results for the group-based MANCOVA including MDD and GAD diagnostic status as fixed factors by question (questions are paraphrased). Bolded numbers in the MANCOVA table indicate significance.

|  |  | **Outcome Expectations** | | | | | **Outcome Values** | | | | | | | |  |
| --- | --- | --- | --- | --- | --- | --- | --- | --- | --- | --- | --- | --- | --- | --- | --- |
|  |  | **Guilt** | **Victim Suffering** | **Dominance** | | **Forced Respect** | | **Punishment** | | **Victim Suffering** | | **Dominance** | | **Forced Respect** | |
| **Results for ICU and ARI of MANCOVA involving ICU and ARI raw score covariates** | | | | | | | | | | | | | | |  |
| **Group** | *F* | **26,32** | 3.34 | 0.721 | 0.005 | | **19.755** | | **23.351** | | 0.798 | | 0.063 | |  |
|  | *p* | 0.000 | 0.070 | 0.397 | 0.945 | | 0.000 | | 0.000 | | 0.373 | | 0.802 | |  |
|  | pη^2^ | 0.13 | 0.018 | 0.004 | 0.000 | | 0.100 | | 0.116 | | 0.004 | | 0.000 | |  |
